# Reproductive roles of the vasopressin/oxytocin neuropeptide family in teleost fishes

**DOI:** 10.3389/fendo.2022.1005863

**Published:** 2022-10-13

**Authors:** Jan A. Mennigen, Divya Ramachandran, Katherine Shaw, Radha Chaube, Keerikkattil P. Joy, Vance L. Trudeau

**Affiliations:** ^1^ Department of Biology, Faculty of Science, University of Ottawa, ON, Canada; ^2^ Department of Zoology, Institute of Science, Banaras Hindu University, Varanasi, India; ^3^ Department of Biotechnology, Cochin University of Science and Technology, Kochi, India

**Keywords:** endocrine system, hormone, hypothalamic–pituitary–gonadal axis, isotocin, neuromodulator, courtship behaviour, paracrine, vasotocin

## Abstract

The vertebrate nonapeptide families arginine vasopressin (AVP) and oxytocin (OXT) are considered to have evolved from a single vasopressin-like peptide present in invertebrates and termed arginine vasotocin in early vertebrate evolution. Unprecedented genome sequence availability has more recently allowed new insight into the evolution of nonapeptides and especially their receptor families in the context of whole genome duplications. In bony fish, nonapeptide homologues of AVP termed arginine vasotocin (Avp) and an OXT family peptide (Oxt) originally termed isotocin have been characterized. While reproductive roles of both nonapeptide families have historically been studied in several vertebrates, their roles in teleost reproduction remain much less understood. Taking advantage of novel genome resources and associated technological advances such as genetic modifications in fish models, we here critically review the current state of knowledge regarding the roles of nonapeptide systems in teleost reproduction. We further discuss sources of plasticity of the conserved nonapeptide systems in the context of diverse reproductive phenotypes observed in teleost fishes. Given the dual roles of preoptic area (POA) synthesized Avp and Oxt as neuromodulators and endocrine/paracrine factors, we focus on known roles of both peptides on reproductive behaviour and the regulation of the hypothalamic-pituitary-gonadal axis. Emphasis is placed on the identification of a gonadal nonapeptide system that plays critical roles in both steroidogenesis and gamete maturation. We conclude by highlighting key research gaps including a call for translational studies linking new mechanistic understanding of nonapeptide regulated physiology in the context of aquaculture, conservation biology and ecotoxicology.

## 1 Introduction

Members of the arginine vasopressin (AVP)/oxytocin (OXT) molecular family and their receptors are phylogenetically ancient, originating in a common ancestor evolved from a common ancestor of the Bilateria (1). As conserved cyclic nonapeptides, they exert pleiotropic functions in reproduction, sociosexual behavior, energy balance, osmoregulation, and the cardiovascular system, among others. In this review we take a focussed approach and examine their importance in the reproductive physiology of teleost fishes.

### 1.1 The teleost nonapeptide system

#### 1.1.1 Nonapeptide structure and evolution

In basal cephalochordates, such as the Florida lancelet, *Branchiostoma floridae*, and urochordates such as the sea vase, *Ciona intestinalis*, single nonapeptides, [Ile^4^]-VP and Ciona (ciVP) have been reported (2–4). The presence of a single AVP family peptide extends to basal vertebrate agnathans, including lampreys such as the Japanese lamprey, *Lethenteron japonicum*, and hagfishes such as *Eptatretus burgeri* (5, 6). It is believed that two rounds of whole genome duplication (2R WGD), one before and one after the separation of agnathans and gnathostomes (7), led to a duplication of the ancestral arginine vasotocin (*avp)* gene. This duplication gave rise to two nonapeptide families conserved in vertebrates; the AVP and OXT family peptides (8). In vertebrates, both genes code for precursor proteins which include a 5’ signal sequence, a highly conserved nonapeptide and neurophysin, and, in the case of the AVP family, a C-terminal glycoprotein termed copeptin without known biological function (9). Processing into the mature nonapeptides occurs *via* prohormone convertases, and the acidic neurophysins I (OXT family) and neurophysin II (AVP family) associate with mature nonapeptides acting as carrier molecules within the neurosecretory system (9).

In teleost fishes, vasotocin (Avp) was originally isolated and characterized in pout, *Gadus luscus* (10), pollock, *Polacchius virens* (11), and European hake, *Mercluccius mercluccius* (12), and has since been identified in genomes of all teleost fishes studied to date (3, 6). Avp occurs in all verebrates except for mammals, where it appears to have mutated to give rise to vasopressin (AVP) in which ^3^Ile was substituted for ^3^Phe (3, 5, 6). All AVP family members are basic nonapeptides due to a basic amino acid (AA) at position 8.

In contrast to the AVP family peptides, the duplicated nonapeptide gene appears to have accumulated more mutations giving rise to the OXT family (3, 5, 6). The OXT nonapeptides are characterized by a neutral AA at position 8 (Leu, Ile, Gln or Val). In fishes alone, as many as 12 Oxt family peptides are known and likely arose due to lineage-specific duplications followed by substitutions in AA positions 3, 4 and 5 in the ring structure and position 8 in the tail structure (**Table 1**). With reference to mammalian OXT, the substitutions are Tyr^2^ by Phe^2^, Ile^3^ by Phe^3^, Gln^4^ by Ser^4^ or Asp^4^, and Leu^8^ by Ile^8^ or Val^8^. In teleost fishes, a single OXT family peptide (Oxt; [Ser^4^-Ile^8^]-OXT) was first isolated in pout, pollock, and the European hake and originally termed isotocin due to the presence of Ile in position 8 (18).

**Table 1:** Oxt nonapeptide family orthologues in different fish groups.

Species/Groups	Historical name	AA sequence	Reference
Holocephali: *Callorhinchus milli* *Hydrolagus colliei*	Oxytocin	CYISNCPQG	(3)
Skates: *Raja miraletus*	Glumitocin	CYISNCPQG	(8)
Sharks: *Squalus acanthias*	Aspargtocin Valitocin	CYINNCPLG CYIQNCPVG	(13)
*Scyliorhinus caniculus*	Asavatocin Phasavatocin	CYINNCPVG CYFNNCPVG	(14)
*Triakis scyllium*	Asavatocin Phasitocin	CYINNCPVG CYFNNCPIG	(15)
Rays: *Torpedo marmorata*	Isotocin b	CYISNCPIG	(16)
Spotted gar: *Lepisosteus oculatus*	[Phe^2^, Ser^4^]-Oxytocin (a variant of ITb) Isotocin b	CFISNCPIG CYISNCPIG	Genomic sources (6)
Most teleost fishes	Isotocin b	CYISNCPIG	(8)
Catfishes: *Heteropneustes fossilis, Clarias batrachus*	Isotocin a (Sevatocin) Isotocin b	CYISNCPVG CYISNCPIG	(6)
Some lungfish, Coelacanth, *Latimeria chalumnae*	Mesotocin	CYIQNCPIG	(17)
Australian lungfish, *Neoceratodus forsteri*	[Phe^2^]-Mesotocin	CFIQNCPG	(8)
*Human* *Homo sapiens*	Oxytocin	CYIQNCPLG	

The AA sequences of the mature hormones are given. Coloured and underlined AA are modifications from the mammalian OXT AA sequence.

**Figure 1:**
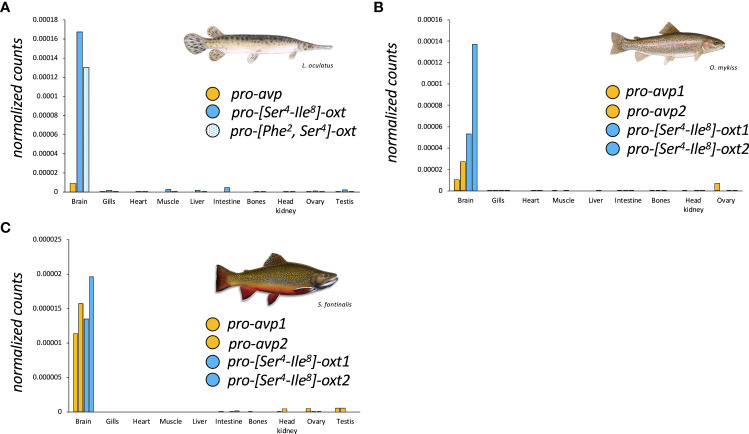
Tissue-specific expression profiles of nonapeptide precursor coding genes in spotted gar **(A)**, rainbow trout*, Oncorhynchus mykiss* **(B)**, and brook trout, *Salvelinus fontinalis* **(C)** derived from the Phylofish RNA-seq database (www.phylofish.signae.org) queried on June 1^st^, 2022 (20).

While position 8 mutations are very common in cartilaginous fishes (19) specific Oxt family peptides have also been reported in Sarcopterygii, such as the Australian lungfish, *Neoceratodus forsteri*, which expresses [Phe^2^-Ile^3^] OXT (20). Based on more recent genomic data, it has become evident that non-teleost Actinopterygians also express specific Oxt peptides (6). Indeed, in the spotted gar, *Lepisosteus oculatus*, which possess a pre-3R genome, two gene paralogues coding for Oxt were identified - one coding for a pro-[Ser^4^-Ile^8^]-OXT with a long C-terminal (NCBI Accession No. XM_006626499.1) and the other coding for a novel pro-[Phe^2^, Ser^4^]-OXT, which has a short C-terminal like other vertebrate neutral OXT family peptide precursors (NCBI Accession no. XM_006626523.1). An analysis of tissue expression profiles based on a fish RNA-seq expression database (21) does not reveal clear-cut differential expression of both *oxt* paralogues in spotted gar (**Figure 1A**).

In teleosts, characterized by a 3R genome condition, occurrence of two copies each of pro-*avp* (pro-a*vp*1 and pro-*avp2*) and pro-*oxt* (pro-*oxt*1 and pro-*oxt*2) have been reported in the blind cave fish, *Astyanax mexicanus*, based on genomic information and in salmonids and catastomids based on cloning studies (6, 22–24). In the blind cave fish, a diploid fish, synteny analysis suggests that the gene duplications may be due to 3R without subsequent gene loci losses (6). In salmonids and catostomids, the multiple *oxt* copies may be due to tetraploidization (4R WGD) and/or gene conversion (22–24). Regarding tissue expression profiles of paralogous *oxt* genes in salmonids, analysis of RNA-seq data (21) suggests similar expression profiles, at least in, *rainbow trout, Oncorhynchus mykiss* (**Figure 1B**), and brook trout, *Salvelinus fontinalis* (**Figure 1C**).

More recently however, the paradigm that teleost fishes, while encoding multiple gene loci for neutral nonapeptides in some cases, nevertheless exclusively express Oxt ([Ser^4^-Ile^8^]-OXT) was challenged by the cloning and discovery of two different neutral Oxt family peptides in the Asian stinging catfish, *Heteropneustes fossilis*, and the walking catfish, *Clarias batrachus* (6). In both species, a conventional *oxt* gene coding for a [Ser^4^-Ile^8^]-OXT and a second *oxt* gene coding for the novel [Ser^4^-Val^8^]-OXT, coined sevatocin, are expressed in addition to a single *avt* gene (6). Like pro-[Ser^4^-Ile^8^]-OXT, the peptide precursor encoding for [Ser^4^-Val^8^]-OXT has a similar organization (**Table 2**), with an extended C-terminal and a Leu-rich region. The functional implications of the *oxt* genes in these catfish species deserve special mention. The substitutions in the hormone moiety (Ile^8^/Val^8^) may lead to altered receptor‐ligand interaction with possible sub- or neo-functionalization. Both *oxt*-like genes are similarly expressed in the preoptic area (POA), and functional studies show that both synthetic [Ser^4^-Ile^8^]-OXT and [Ser^4^-Val^8^]-OXT similarly regulate *fshb, lhb* and *gpa* expression in the catfish pituitary (6, 24). It is possible that both Oxt peptides bind to the same receptors, but receptor characterization has not yet been undertaken.

**Table 2:** General features of the cDNAs of an encoded precursor proteins of Oxta, Oxtb and Avp in the catfish Heteropneustes fossilis.

Nonapeptide	cDNA (bp)	Coding sequence (bp)	Precursor protein (AAs)	Signal peptide(AAs)	Neurophysin peptide (NP)(AAs)	Cys in NP(AA)	Leucine-rich core
Oxta	619	1-462	153	19	122	14	LLRKLLHL
Oxtb	708	56-508	151	29	118	14	LLKLLHL
Avp	618	60-524	155	20	122	14	LLLRILH

The mature nonapeptide hormone moiety (9 AA), cleavage site (GKR), and the domain between signal peptide and neurophysin are not indicated.

For the purpose of this review, we follow the recent Zebrafish Information Network (ZFIN, www.zfin.org) nomenclature, which uses a*vp/*Avp and *oxt/*Oxt to designate teleost genes and their protein products. The reasoning for this recent change in the literature is to highlight the homologous nature of *avp* and *oxt* family genes and their products in vertebrates. Consequently, this nomenclature no longer uses the historical distinction between teleost and mammalian nonapeptides (arginine vasotocin/arginine vasopressin and isotocin/oxytocin) which is reflective of their AA composition. Thus, while the historically widely used nomenclature is not used in this review, it is implicitly understood that the teleost *avp*/Avp and *oxt*/Oxt differ from mammalian and other vertebrate nonapeptide homologues in their AA residues as described.

#### 1.1.2 Anatomy of the nonapeptide system in the context of reproduction

The distribution of Avp and Oxt has been examined in many teleosts and will not be covered in detail here. Controversies arise largely because of differing sensitivities of the neuroanatomical methods used, variable control experiments and other technical challenges (25, 26). Nevertheless, the use of transgenic approaches is helping to firmly establish the key locations of neuronal soma, and a new appreciation for their wide projection fields (27–30). In teleosts, Avp and Oxt neurons are intermingled and have been classified into three populations in the POA based on soma size. These are the giganto-, magno- and parvocellular neurons. Preoptic Avp and Oxt neurons send their fibers into diverse regions of the brain such as the hypothalamus, ventral telencephalon, mesencephalon and diencephalon, as well as the hindbrain and the spinal cord (20–30, amongst many others). Through the latter two systems, a role for POA-derived nonapeptides in modulating motor output related to reproductive behaviour and gamete release has been postulated in some, but not all teleost species (31–33).

In fish species in which specific sensory modalities have been shown to play a key role in reproductive behaviours, nonapeptide innervation has been described for distinct brain regions involved in sending and receiving sensory information. For example, in male zebrafish, *Danio rerio*, known to respond to female sex pheromones (34), fibers positive for Oxt have been identified in the olfactory bulb (35). In the plainfin midshipman, *Porichtys notatus*, which relies on vocalization as part of their courtship behaviour (36), especially Avp but also Oxt innervation was found in fore- and mid-brain regions involved in vocalization, and diencephalic regions of the ascending auditory pathway (37, 38). In the weakly electric gymnotiform bluntnose knifefish, *Brachyhypopomus gauderio*, which uses electric organ discharge (EOD) signals for mate selection (39), Avp innervation in the medulla was found to be in proximity of the pacemaker nucleus that controls EOD (40). Together, neuroanatomical evidence thus suggests a role for nonapeptides in modulating both emitting and receiving pathways of diverse sensory signals linked to reproductive behaviours in various teleost fishes.

In all teleost species studied to date, prominent preoptic and ventral hypothalamic projections terminating in the posterior pituitary for release to the pituitary vasculature have been reported (41, 42). In some species, such as the dwarf gourami, *Colisa lalia*, a colocalization of parvocellular Oxt with gonadotropin releasing hormone (Gnrh) has been reported (43). In the goldfish, *Carassius auratus*, Oxt colocalizes with secretoneurin a (44), an important hypophysiotropic stimulator of the hypothalamo-pituitary-gonadal (HPG) axis (45, 46). Such neuroanatomical data suggest that nonapeptides may regulate the HPG axis by co-release with other neuropeptides.

Detailed neuroanatomical studies of the pituitary in the sailfin molly*, Poecilia lattipinna*, the European bass, *Dicentrarchus labrax*, and the African sharptooth catfish, *Clarias gariepinus*, revealed that nonapeptidergic innervation principally forms contact with pituitary vasculature in the form of terminal release buttons which highlights an endocrine role of the nonapeptides (41, 42, 47, 48). Central and circulating Avp and Oxt concentrations in the nM range have been reported to be sex-specific, correlated, and reproductive stage-dependent in at least some species, such as the round goby, *Neogobius melanostomus* (49), and the air-breathing catfish, *Heteropneustes fossilis* (50). In addition to pituitary release, nonapeptide fibers innervating gonadotrophs in the proximal pars distalis through gaps in the basement lamina have been demonstrated in the sailfin molly, *Poecilia latipinna* (41, 48). While there are far fewer contact sites of nonapeptide innervation of gonadotrophs compared to pituitary blood vessels, this evidence does nevertheless suggest that the neuronal organization of nonapeptide fibers in the pituitary also provides a basis for paracrine effects on gonadotrophs. Whether a single nonapeptide neuron originating from the teleost POA can be both encephalotropic and hypophysiotropic, and thus simultaneously regulate brain function as a neuromodulator and peripheral function as a paracrine factor or hormone, remains an open question. Evidence to-date suggests that this may be species-dependent. While single nonapeptide neurons originating in the POA have been shown to extend to both extrahypothalamic brain regions and the pituitary in Atlantic salmon, *Salmo salar* (51), clearly distinct Avp and Oxt neurons originating in the POA have been shown to innervate either extrahypothalamic brain regions or the pituitary, but not both, in zebrafish, *Danio rerio* (28, 29).

Transcript and/or protein abundance of nonapeptides in female and, to a lesser extent, male gonads has also been reported in several species. In rainbow trout, *Onchorhynchus mykiss*, ovarian expression of both *avp* and *oxt* has been reported (52). High *oxt* expression is also noted for whole zebrafish ovaries (53). In the air-breathing catfish, high-performance liquid chromatography (HPLC) analysis indicated the presence of Avp in ovaries and, albeit to a much lower extent, in testes (50). Immunohistochemistry approaches localized Avp to the ovarian follicular cell layer, with positive staining in both theca and granulosa cells, but failed to locate Avp in testes (50). Conversely, Avp has been located to interstitial cells in the testes of the chanchita, *Cichlasoma dimerus* (54). The expression of gonadal nonapeptide systems, and especially *avp*, appears to be a more widespread feature in teleosts, as suggested by RNA-seq data mined from the Phylofish database (20) and presented here (**Figures 1A-C**). Together, evidence of expression of a gonadal nonapeptide system in female and male gonads provides the anatomical basis for an additional reproductive role of nonapeptides thorough paracrine modulation of processes such as gametogenesis, steroidogenesis, and gamete release.

### 1.1.3 The teleost nonapeptide receptor repertoire

Nonapeptide systems can regulate reproductive physiology *via* central neuromodulatory and/or peripheral endocrine and/or paracrine pathways *via* G-protein coupled membrane**-**bound AVP and OXT peptide family receptors (AVPRs and OXTRs). Since newly available genome sequences have recently allowed for a comprehensive description of the vertebrate nonapeptide receptor repertoires and their evolution resulting in proposed nomenclature changes (55–59), we here review the repertoire of teleost receptors considering these findings in detail. For the purpose of this review, we once again follow the ZFIN nomenclature, which is largely reflective of these changes. This focused review provides the basis to critically review relevant information regarding specific teleost Avpr and Oxtr function in mediating central and peripheral nonapeptide effects on teleost reproduction.

**Figure 2:**
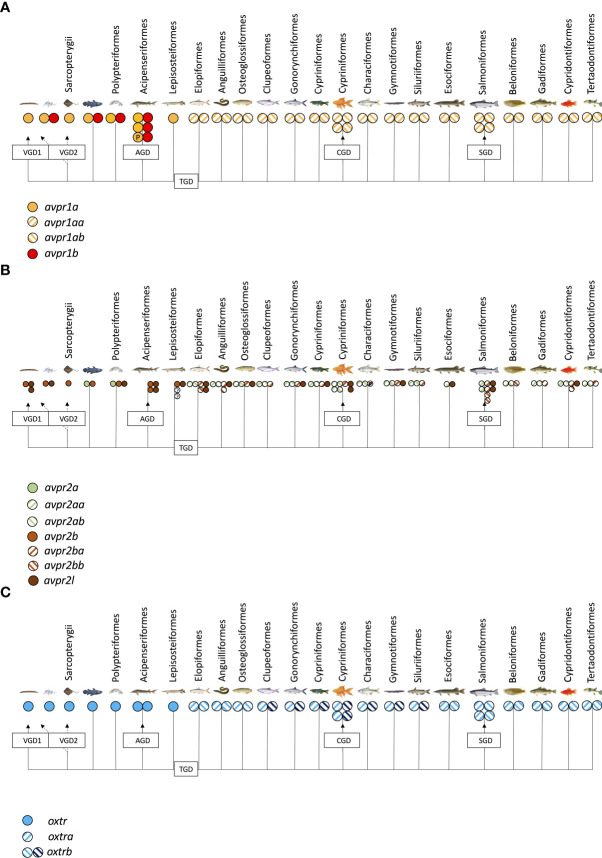
Presence of *avpr1* **(A)**, *avpr2/avpr2l* **(B)** and *oxtr* **(C)** genes in published fish genomes accessed on NCBI (www.pubmed.com) on June 1^st^, 2022.

**Figure 3:**
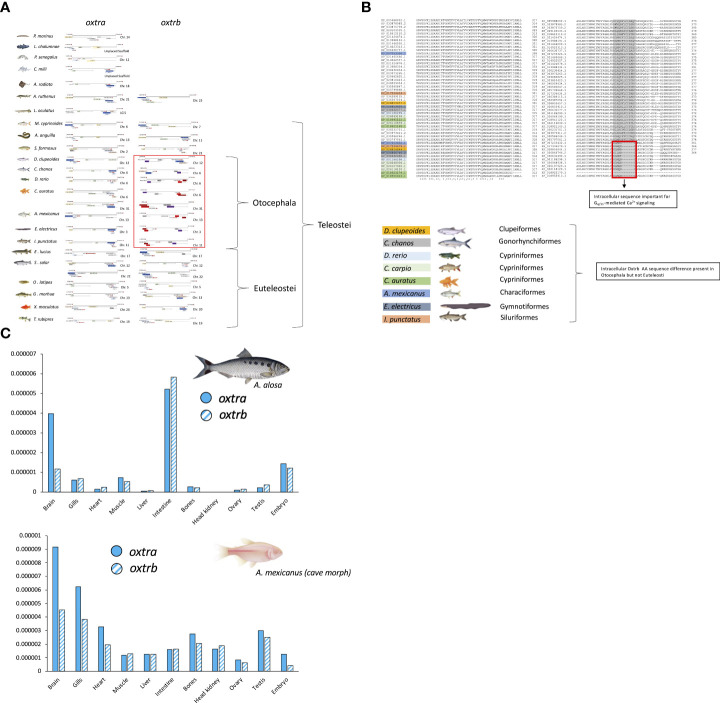
Microsynteny analysis **(A)**, predicted AA sequence alignment **(B)** and RNA-seq based tissue expression profiles **(C)** of *oxtra* and *oxtrb* paralogues in select teleost fishes. Micro-synteny analysis of *oxtr* paralogue gene loci was manually conducted on NCBI (www.pubmed.com) derived genome sequences retrieved on June 1^st^, 2022, while *oxtr* gene paralogue sequence-predicted AA sequences were aligned using Clustal Omega (https://www.ebi.ac.uk/Tools/msa/clustalo/). RNA-seq based tissue expression profiles of *oxtr* paralogues was created derived from the Phylofish RNA-seq database queried on June 1^st^, 2022 (20).

The teleost nonapeptide receptor repertoire consists of five distinct gene family members, although not all are present in every teleost species. These receptors include *avpr1a, avpr2, avprl* as well as *oxtr* and their paralogues. A fifth vertebrate nonapeptide receptor family member, *avpr1b* is present in basal ray-finned fishes as well as tetrapods but is not present in elasmobranchs and teleost fishes (55–59) (**Figures 2A-C**). With regard to intracellular signaling mechanisms, Avprs and Oxtrs use diacylglycerol (DAG), inositol triphosphate (IP_3_) and calcium (Ca^2+^) as second-messengers with the exception of Avpr2l, which uses cyclic adenosine monophosphate (cAMP) (60). Avpr2b signaling has not directly been assessed in teleost fishes but may be similar to Avpr2aa, based on intracellular domain AA sequence similarities (57). For *avpr1*, all teleost genomes analyzed to date possess two paralogues, termed *avpr1aa* and *avpr1ab* (**Figure 2A**). Additionally, all teleost fishes appear to possess *avpr2aa* and *avpr2ab* paralogues (**Figure 2B**). The presence and retention of these paralogues is in line with the teleost-specific genome duplication, as single *avpr1a* and *avpr2a* genes exist in early non-teleost fishes (**Figures 2A, B**). Conversely, the situation appears to be more complex with regard to *avpr2b* and *avpr2l* genes which are retained in different paralogue numbers and lost in some but not all teleost fishes in no obvious evolutionary pattern (**Figures 2A, B**). The *oxtra* and *oxtrb* paralogues are found in all teleosts assessed to date, again indicative of a retention of paralogues following genome duplication at the base of teleost evolution (**Figure 2C**). Teleost-specific changes in the nonapeptide receptor repertoires have been reported, especially regarding possible intracellular AA sequences involved in cell signalling. For example, the teleost Avpr2aa AA sequence harbours extensions of the intracellular loop 3 region, which include relatively well-conserved Tyr, Ser and Thr residues which can be phosphorylated by a variety of intracellular kinases to alter intracellular signaling cascades as well as G-protein coupled receptor attenuation/desensitization, endocytosis, and intracellular trafficking (59). Lineage-specific changes in the nonapeptide repertoires have also been reported even within the infraclass of teleost fishes. For example, otocephalans lack an *avpr2bb* paralogue found in early teleosts as well as euteleosts (59), as shown in **Figure 2B**. In our current microsynteny analysis, we found additional otocephalan-specific changes for the *oxtrb* nonapeptide receptor locus (**Figure 3A**). These microsynteny changes translate into C-terminal AA sequence changes in an intracellular domain which, based on annotation from functional mutation studies in the human OXTR (61), is linked to G_q_-protein recruitment and cell signaling (**Figure 3B**). Otocephala, which diverged from Euteleostei during the Jurassic ~220 million years ago, are characterized by innovations in communication and sensing, notably through Schreckstoff, the alarm substance released through skin injury from conspecifics, and auditory capacity through a link between the swim bladder and inner ear (otophysic link) (62). It is thus tempting to speculate that such rearrangements, with possible functional implications, represent changes which facilitated sub- or neo-functionalization linked to increased social cues relevant to reproduction in otocephalans. In the case of otocephalan Oxtrb, a loss of highly conserved intracellular domain AA residues was observed (**Figure 3B**). These are specifically located in the C-terminal sequence and have been shown to be required for G_q/11_ and subsequent IP_3_, DAG and Ca^2+^ signaling (61). They also include consecutive Cys residues, which through palmitoylation, have been shown to anchor the C-terminal region to the membrane (61). Finally, it includes consecutive Ser residues believed to be involved in receptor retention for receptors following ligand-dependent internalization (61). Investigation of *oxtra* and *oxtrb* expression using an RNA-seq-based fish tissue database (21) reveal that in the otocephalans Allis shad, *Alosa alosa*, and Mexican cavefish, expression pattern for both *oxtra* and *oxtrb* receptor paralogues are similar in most tissues, apart from the brain where *oxtra* expression exceeded *oxtrb* expression (**Figure 3C**). In zebrafish ovary, *oxtrb* appears to be more strongly expressed compared to *oxtra* (53), suggesting possible differential roles in regulating ovarian function (53).

In addition to *oxtr* paralogues, the expression of other teleost nonapeptide receptors and in some instances specific paralogues have been localized to tissues relevant to teleost reproduction (63–73) and are summarized in **Table 3**. Much of our current information on reproductive roles of nonapeptides has been derived from studies of otocephalan species including the air-breathing catfish (4, 5, 24, 84), goldfish (85–87), and zebrafish (35). Given that the nonapeptide repertoire is characterized by genomic rearrangements and potentially important AA sequence changes in this clade, caution is warranted when attempting to generalize functionality across all teleosts.

**Table 3:** Nonapeptide receptor expression in central and peripheral teleost tissues relevant to reproduction.

Tissue	Receptor type	Species	Description	Reference
Brain	*avpr1aa* *avpr1ab* *avpr2ab*	Air-breathing catfish *Heteropneustes fossilis*	*avpr1aa* type receptor mRNA is expressed in major neuroendocrine hypothalamic and telencephalic nuclei including the POA and sensorimotor centres; *avpr2ab* type receptor mRNA is largely confined to subependymal telencephalon	(74, 75)
	*avpr1aa* *avpr1ab* *avpr2aa* *avpr2ab* *oxtrb*	Pupfish, *Cyprinodon nevadensis amargosae*	*avpr1aa*, *avpr1ab*, *avpr2ab* and *oxtr* mRNA is expressed in telencehalon, hypothalamus and hindbrain	(76)
	*avpr1aa; avpr1ab*	Zebrafish, *Danio rerio*	mRNAs are expressed in forebrain, midbrain, and hindbrain. *avpr1aa* positive hindbrain neurons are contacted by *avp* neurons originating from POA and lateral longitudinal fasciculus and extending to sensorimotor areas such as the medial longitudinal fasciculus	(77)
	*avpr1ab*	Atlantic Croaker	mRNA and protein localized to hypothalamic GnRH neurons	(78)
	*avpr1ab*	Rock hind, *Epinephelus adscensionis*	mRNA is widely distributed in brain areas linked to reproductive and sensorimotor control including hypothalamic GnRH neurons, POA and olfactory bulb	(63, 79)
	*avpr1aa*; *oxtra*	*Astatotilapia burtoni*	mRNA and protein expressed in telencephalon and hypothalamus	(80)
**Pituitary**	*avpr1aa*; *avpr1ab*; *avpr2ab*	Air-breathing catfish *Heteropneustes fossilis*	mRNA expressed in male and female rostral pars distalis and pars nervosa	(74, 75)
	*oxtra*	Rice-field eel *Monopterus albus*	mRNA located to Lh but not Fsh cells	(81)
**Gonad**	*avpr1aa*; *avpr1ab*; *avpr2ab*	Air-breathing catfish *Heteropneustes fossilis*	In testes, *avpr1ab* and *avpr2ab* receptor mRNA are localized to interstitial tissue seminiferous epithelium. In ovaries, *avpr1aa* and *avpr1ab* receptors are localized to the follicular layer and an *avpr2ab* receptor to the oocyte membrane	(74, 75)
	*oxtra*	Guppy	Ovaries, expressed in follicular layer	(82)
	*avpr1aa*; *avpr1ab*	Bluehead wrasse	Ovaries, Testes	(83)

In contrast to the detailed evolutionary history, the functional characterization of nonapeptide receptors remains limited, and in some instances, such as for *avpr2l*, virtually unexplored in teleost fishes. Early studies in a cyprinid, the white sucker, *Catostomus commersonii*, investigated binding kinetics and specificity of both Avpr (88, 89) and Oxtr (90). Based on teleost genome sequences available today, these can retroactively be classified as Avpr1ab and Oxtra, respectively. These studies revealed that the Avpr1ab is highly selective for Avp (EC_50_ 13 nM ± 6 nM) over Oxt. Both nonapeptides bound to and activated cell signaling of a heterologously expressed Oxtra receptor with 3- to 4-fold higher affinity for Oxt (80 ± 30 nM) compared to Avp (300 ± 90 nM). More recent studies in zebrafish assessed specificity of nonapeptides and a mammalian OXT receptor antagonist (L-368,899) in heterologous expression assays (91). It was reported that Oxt exhibits similar affinities for both Oxtr parlogues with EC_50_ values of 2.99 ± 0.93 nM for Oxtra and 3.14 ± 1.10 nM for Oxtrb, respectively. In comparison, Avp exhibited slightly lower affinities to Oxtrs with EC_50_ values of 11.0 ± 3.0 nM for Oxtra and 27.0 ± 9.5 nM for Oxtrb, respectively. Both Avp and Oxt had low affinities for Avpr1aa (EC_50_ 727 ± 338 nM and 317 ± nM, respectively) and high affinities for Avpr1ab (2.79 ± 1.4 nM and 3.52 ± 0.94 nM, respectively). The guppy Oxtra was shown to be activated by both Oxt and Avp (82). While Oxt induced a strong dose-response (10 nM-1 μM) induction of co-transfected luciferase CRE-element, Avp induced a weaker, yet significant response at all concentrations tested (82). Together these data show that at physiological concentrations, it is likely that some degree of cross activation between Avp and Oxt ligands and the nonapeptides receptors occurs. It is therefore important to consider that reproductive roles of Avp and Oxt may, at least in part, be dependent on receptor cross-activation.

## 2 External and internal reproductive cues regulate teleost central nonapeptide systems

In several seasonal teleost fishes, nonapeptide expression and/or protein abundance in the POA is positively correlated with mature reproductive status (49, 50), which in turn, is dependent on environmental cues such as photoperiod and temperature (92). In female goldfish, hypothalamic *oxt* mRNA abundance peaks in the seasonal breeding period when fish have maximal gonadosomatic index (GSI) and is likely photoregulated (93). In male goldfish, exposure to the female releaser pheromone PGF_2α_, an important olfactory reproductive cue for males, increases *oxt* and *avp* mRNA levels in the telencephalon while stimulating circulating testosterone (T) concentrations and strippable milt volumes (94). Together, such association of nonapeptide expression in the neuroendocrine brain and enhanced pituitary and gonadal activity during seasonal cycles and after pheromone exposure is supportive of a physiological role in reproduction. Exposure to androstenedione, another pheromone known to play an important role in male-male competition in a reproductive context in goldfish (95), increases parvocellular *avp* (96). Thus, it appears that reproductive pheromones represent important environmental cues to regulate male goldfish nonapeptide expression. In male and female round goby, *Neogobius melanostomus*, maximal seasonal brain Avp concentration was observed just before spawning in March-April, whereas that of Oxt peaked during spawning in May-June (97). The lowest brain Avp level was noted in the non-breeding season from November to January, while the level of Oxt decreased immediately at the end of the spawning. The results show that high Avp levels correlate with pre-spawning period whereas the highest Oxt levels correspond to spawning. In female round gobies, these increases appear, at least in part, to be dependent on estrogens acting *via* genomic (nuclear receptors), and genomic and non-genomic mechanisms in the case of Avp and Oxt, respectively (98). This suggests that season-dependent gonadally-derived positive sex steroid feedback mechanisms may reinforce the seasonal activation of hypothalamic nonapeptide systems. In the half-spotted goby, *Asterropteryx semipunctata*, Avp and Gnrh protein abundances were positively correlated and exhibited significant peaks in their abundances in sexually mature females (99). In male sticklebacks, the highest brain concentrations of Avp were observed in the most aggressive males that cared for eggs and nuptial-colored subordinates that fought to change their social status. Oxt was significantly higher in brains of aggressive dominant males (100). In the stickleback, the highest Avp levels were found in brains of females that did not deposit eggs, regardless of whether they were kept with courting or non-courting males and whether they had a nest or not. The highest Oxt levels were observed in females that did not deposit eggs but were kept with a courting male. The presence of courting or non-courting males that somehow activate Oxt- or/and Avp-producing neurones may be decisive for both behaviour and/or final oocyte maturation or egg deposition, because brain levels of both nonapeptides decreased sharply after egg deposition (101).

In the anadromous chum salmon, *Oncorhynchus keta*, dynamic changes in nonapeptide transcript and protein abundance have been reported during the reproductive migration period (102, 103). Lower transcript but higher immunoreactivity of both Oxt and Avp in the POA of fish collected upstream in a freshwater system compared to those caught in the marine bay, which serves as an entry point of the reproductive migration (102, 103). Based on these results it is tempting to speculate that due to their well-described role in osmoregulation, nonapeptide changes may act to integrate relevant environmental signals, such as the change in salinity, to subsequently change aspects of reproductive physiology in these anadromous fish. It has, however, been shown that salinity changes are not consistently the principal factor mediating changes in the nonapeptide systems of migrating salmonids and temperature and endogenous sex steroid concentrations also act as important regulators of these systems (104). Nevertheless, these data demonstrate a reproductive phase-dependent regulation of the nonapeptide system in chum salmon. Within the context of salmonid reproduction and migration, it is important to note that alternative reproductive tactics exist among individuals. In addition to males migrating to the ocean to grow and mature, some precocious parr achieve sexual maturity quickly before migrating to the ocean and can thus fertilize female eggs as ‘sneaker males’ (105, 106). Two transcriptomic and targeted gene expression studies investigating differential gene expression in the whole brain of maturing males and precocious parr of Atlantic salmon identified nonapeptides as being differentially regulated (107). The functional relevance of the differential expression with regard to reproduction remains, however, unknown.

In some group-living cichlid species in which HPG axis activity and reproductive behaviour is linked to social dominance, *avp* and Avp abundance has been investigated (108). The results show differential effects on *avp* mRNA levels and Avp neuron size in different POA subpopulations, with higher levels in gigantocellular neurons and, conversely, lower levels in parvocellular neurons of dominant fish. While underlining the responsiveness of the nonapeptide system to social status, a determinant of reproductive status in at least some cichlids, these findings also highlight the importance of considering potentially differential effects on nonapeptide subpopulations in the POA. However, the finding that *avp* expression in the POA of dominant and reproductively active African cichlid fish, *Astatotilapia burtoni*, is significantly reduced compared to subordinates who are reproductively supressed (109), clearly suggests caution is warranted to avoid simplified and global paradigms regarding the role of nonapeptides in species in which social dominance is linked to increased reproductive capacity.

Teleost fish exhibit significant plasticity in terms of their reproductive biology and life history traits. Some species are sequential hermaphrodites, beginning life as one sex, and changing sometime later to the other. Such species are capable of protandrous (male-to-female), protogynous (female-to-male), or serial (bidirectional) sex change (110). Changes in nonapeptide systems have been observed in response to social context-induced sexual plasticity. For example, the bluehead wrasse, *Thalassoma bifasciatum*, exhibits specific increases in Avp immunoreactivity in the magnocellular POA when undergoing behavioural female-to-male sex change that occurs rapidly following the removal of a large terminal colour male and is independent of the gonads (111). The increase in magnocellular *avp* expression coincides with a rapid increase in dominant and male courting behaviour, suggesting a functional link (111, 112). Serial adult sex change in the marine goby, *Trimma okinawae*, is associated with significant and reversible changes in the size of Avp-producing forebrain cells, which are higher in males and coincide with increased male mating behaviour (113). In overcrowded single sex groups of female black mollies, *Poecilia sphenops*, which do not form social hierarchies, masculinization of reproductive behaviour occurs and is linked to a decrease of higher, female-typical Avp concentrations in this species to male typical concentrations (114). In a transcriptomic analysis of the bluehead wrasse forebrain, increased *oxt* transcript abundance was one of the few statistically significant changes detected across female-to-male sex change (115). The opposite trend was observed in the bluebanded goby, *Lythrypnus dalli*, where lower Oxt immunoreactivity was observed in the POA of males and late-stage female-to-male sex-changers compared to females (116).

Overall, these studies reveal context-dependent regulation of central nonapeptide systems across several species with diverse reproductive strategies. This strongly implicates nonapeptides in the regulation of teleost reproductive physiology. However, caution is clearly warranted to avoid oversimplified paradigms for specific roles of nonapeptides across all teleost species (117), as at least some species-specific roles are likely to have evolved among teleosts with such varied reproductive strategies.

## 3 Reproductive function of nonapeptides in teleost fishes

### 3.1 Nonapeptide-dependent regulation of reproductive behaviour

The plainfin midshipman fish, *Porychthys notatus*, displays sex- and morph-specific vocalization during mating. Oxt and Avp regulate these sex- and morph-specific effects on the vocal circuitry (37). Type I males mating call are stimulated by Avp, whereas female and type II males’ grunting sounds are stimulated by Oxt. Using homozygous Japanese medaka, *Oryzias latipes*, knockout mutants, an essential role for Avp in male mate-guarding behaviours in this non-monogamous species has been demonstrated (118). For example, under natural conditions, two medaka males kept in triads with a female are in competition and the dominant medaka male that is ‘guarding’ the female exhibits increased reproductive success measured as increased paternity in offspring. Males harbouring mutations in a*vp* and *avpr1aa* exhibit significantly reduced male guarding behaviour indicating a key role for *avp* in dominant status-dependent reproductive success (118). Similarly, *oxt* and *oxtra*, exert sex-specific effects in Japanese medaka: mutant female fish exhibit a lack of mate preference for familiar males and mutant male fish have reduced courtships displays to unfamiliar females, but exhibit increased mate-guarding behaviour towards familiar females (64). Since the potential effects on the HPG axis were not quantified in these studies, it is not clear whether these effects are entirely mediated by the nonapeptides, or whether altered HPG axis regulation also contributes to the behavioural observations.

In male bluehead wrasse, *Thalassoma bifasciatum*, a species with alternate male reproductive tactics (territorial and non-territorial), Avp intraperitoneal injection increased courtship behaviour in the field irrespective of male reproductive tactic and promoted a territorial-like phenotype in non-territorial males (65). An opposite effect was observed following administration of Manning’s compound, a mammalian AVPR1 receptor antagonist, suggesting that this effect is mediated *via* this nonapeptide receptor subtype (65). In male white perch, *Morone americana*, intracerebroventricular but not intraperitoneal administration of Avp significantly stimulated an important courtship behavior termed ‘attending’ without affecting whole body or circulating androgens (119). These data suggest that central rather than peripheral HPG axis actions are involved in mediating the effects of Avp on the male white perch courtship behaviour. In male beaugregory damselfish, *Stegastes leucostictus*, Manning’s compound significantly lowered male courtship behaviour, while exogenous Avp administration did not affect male courtship behaviour (120). Similarly, administration of Manning’s compound significantly reduced male reproductive courtship behaviour and reproductive success in mating assays with female zebrafish, *Danio rerio* without affecting whole body androgen (T and 11-keto-testosterone; 11-KT) levels, suggesting Avp acutely regulates male zebrafish courtship behaviour including chasing, nudging, and circling *via* central Avpr1a receptors and independently of HPG axis regulation (35). In the weakly electric fish, *Brachyhypopomus gauderio*, male courtship behaviour observed in male-female dyads resulted in a higher degree of Avp neuron activation in the nucleus preopticus ventricularis anterior compared to isolated males (121). In the same species, Avp increases dominance in part *via* direct modulation of the EOD rate (122). Together, these findings raise the possibility that male reproductive behaviour *via* electric signaling may be under Avp control in this species. Despite the reviewed evidence, a universal Avp-dependent stimulation of male reproductive behaviour in teleost fishes is unlikely, as the reproductive phenotype of females and sneaker males, but not dominant males, is sensitive to Avp in the peacock blenny, *Salaria pavo* (123). There is a need for detailed comparative studies of the roles of nonapeptides in teleosts which exhibit the most diverse reproductive strategies and behaviours amongst the vertebrates (117).

Several studies have provided evidence for regulatory roles of nonapeptides in teleost species with parental care. In the primarily paternal teleost the common clownfish, *Amphiprion ocellari*s for example, administration of an OXTR antagonist abolished paternal behaviours such as nips, fanning the eggs, and proportion of time in the nest, without affecting aggressive behaviours in paired non-reproductive fish (124). This suggests a specific action of Oxt in controlling male common clownfish parental care behaviours (124); however, whether the high selectivity of antagonist (desGly-NH2-d(CH2)5[D-Tyr2,Thr4]OVT) for mammalian OXTR also applies to teleosts has not been formally investigated. When introducing domino damselfish, *Dascyllus trimaculatus*, as non-conspecific intruders, administration of the OXTR antagonist reduced paternal care behaviour in clownfish, but increased aggression towards the non-conspecific intruder, demonstrating the importance of social context in behavioural responses (125). Conversely, administration of an AVTR1 receptor antagonist increased male parental behaviours while reducing aggression towards intruders (125), thus demonstrating antagonistic roles of the nonapeptide systems in common clownfish (124, 125). These data suggest a role for nonapeptides in paternal care. In the monogamous convict cichlid, *Amatitlania nigrofasciata*, single fathers increase paternal care behaviours quickly after removal of the female partner, and this increase coincides with increased activation of parvocellular Oxt neurons in the POA (126). Administration of a mammalian OXTR antagonist in biparental males inhibited paternal care behaviour, indicating a functional role for Oxt neurons (126).

### 3.2 A role for central nonapeptides in regulating saliency to reproductive cues in teleost fishes

In addition to the direct modulation of teleost reproductive behaviours, central roles for nonapeptides may not be restricted to the role of transducing and integrating reproductive cues, but also to act as a filter and/or amplifier of exogenous or endogenous cues. This latter concept has recently gained more traction as the ‘salience hypothesis’, especially in higher vertebrate species, including humans. The salience hypothesis is based on the notion that an individual is being constantly inundated with sensory information in its environment and therefore needs to be able to filter the information to identify what is relevant and important (i.e., signal) from that which is unimportant (i.e., background noise). The nonapeptides OXT and AVP have been proposed to serve as two important neuromodulators in the central nervous system that can help to increase the salience of sensory information, such as conspecific olfactory cues, to induce effects on endocrine and behavioural responses (127). In fish, however, this concept remains comparatively poorly explored, especially in the context of the diverse reproductive strategies in different environmental conditions and cues. Olfactory stimuli in particular play important roles in at least some groups of teleost fish such as cyprinids (35, 94, 95). Future studies exploring sensitizing roles of nonapeptides to reproductive pheromonal and visual cue detection are thus clearly warranted. A recent study in zebrafish identified Oxt-like innervation in the male olfactory bulb (35), providing a neuroanatomical basis for functional studies. Similarly, the widely studied electrical and vocal communication systems in some teleost fishes (128) should provide excellent models to investigate possible modulatory roles of nonapeptides in the reception and integration rather than production of reproductive signals.

### 3.3 Nonapeptide-dependent regulation of the HPG axis

#### 3.3.1 Nonapeptides are an integral part in the hypothalamic circuitry controlling the HPG axis

Both stimulatory and inhibitory factors regulating the teleost HPG axis have been well described (92). Nonapeptides may affect the HPG axis *via* modulation of stimulatory and/or inhibitory hypophysiotropic systems. To date, little evidence exists for potential roles of nonapeptides in directly affecting Gnrh in teleost fish. In rock hind, *Epinephelus adscensionis*, and in Atlantic Croaker, *Micropogonias undulatus*, Avpr1ab receptors are co-localized with Gnrh1 in preoptic anterior hypothalamic neurons. The functional relevance of this crosstalk other than reported concordant regulation of gene expression between *avpr1ab* and *gnrh1* (78, 79, 99) must be investigated. Studies in female goldfish show that serotonin neurons, known to stimulate pituitary Lh release in this species (129), are found in proximity to Oxt neurons in the POA and Oxt fibres in the pars nervosa of the pituitary gland, suggesting a possible interaction between them (87). However, the functional relevance of possible Oxt dependent modulation of serotonin-dependent gonadotropin release has not been investigated.

Nonapeptides may ostensibly also act to promote pituitary gonadotropin release *via* reduction of potent dopaminergic inputs on Lh release (92). This is supported by work in the walking catfish, *Clarias batrachus*, which suggests that Oxt may stimulate Lh release *via* the inhibition of dopaminergic blockage (66). In walking catfish Oxt immunoreactivity was greatly enhanced in the POA in female pre-spawning and spawning fish, and superfusion of brain slices with Oxt resulted in a ~50% reduction of tyrosine hydroxylase staining, suggesting rapid inhibitory effects on dopamine or other catecholaminergic neurons (66).

In addition to hypothalamic interaction between nonapeptide systems and the neuronal circuitry involved in HPG axis regulation in several teleost models (66, 86, 87), a few lines of evidence also demonstrated co-expression of nonapeptides with neuropeptides with known stimulatory function on gonadotropin release. For example, Oxt and secretoneurin were found to be colocalized in the POA and fibers innervating the pituitary in goldfish, *Carassius auratus* (44), while colocalization of Oxt and GnRH was reported in the dwarf gourami, *Colisa lalia* (43). Together, these studies suggest that potential co-release of nonapeptides with other neuropeptides known to stimulate gonadotropin release represent an understudied aspect of HPG axis regulation.

In addition to targeting neuronal circuitry involved in the regulation of the HPG axis, POA nonapeptide neurons in teleost fish themselves receive neuronal input from reproductive neuropeptides. These include Gnrh, which contact Oxt neurons in rainbow trout, *Oncorhynchus mykiss* (130), and kisspeptin, which contact Oxt and Avp neurons in Japanese medaka (131) and striped bass, *Morone saxatillis* (132). Together, this neuroanatomical evidence points to potential neuromodulatory roles for nonapeptides in HPG stimulation in the context of multimodal signaling systems regulating gonadotrophs in teleosts (133). Given the reported roles of nonapeptides in teleost sociosexual and courtship behaviour, neuroanatomical evidence may also reflect synchronization of HPG axis activation stimulation with nonapeptidergic behavioural pathways to maximize reproductive success.

Several studies have also demonstrated the sensitivity of both Oxt and Avp neuronal populations to sex steroids. Administration of both low (0.1 μg/g body weight) and high (0.5 μg/g body weight) estradiol (E_2_) doses (0.5 μg/g body weight) normalizes ovariectomy-induced decreases in brain and plasma Avp concentrations in Asian stinging catfish (134). This effect appears to be, at least in part, indirectly mediated *via* the modulation of dopaminergic control, as treatment with α-methyl-para-tyrosine, a tyrosine hydroxylase inhibitor, partially abolished the restorative effect of the low E_2_ dose on Avp abundance in the ovariectomized fish (134). A male-specific stimulatory effect of androgens on parvocellular Oxt neurons in the medaka POA has also been reported (135). This regulation also appears to be indirect, as androgen receptor expression was not found in Oxt neurons but on kisspeptin neurons known to stimulate Oxt neurons in this species (131, 135). In female round gobies, circulating E_2_ levels are higher in the spawning phase compared to non-spawning phase and coincide with high circulating Avp and Oxt concentrations (98). Brain explant exposure to E_2_ in spawning and non-spawning phases stimulated Avp and Oxt release in this species; however, pharmacological studies using the estrogen receptor (ER) antagonist fulvestrant and the transcription inhibitor actinomycin D showed that the effect of E_2_ on Avp and Oxt release was mediated by different signaling pathways (98). E_2_-dependent Avp release was mediated by ERs *via* both genomic and non-genomic pathways, while Oxt release was mediated through ERs *via* a genomic pathway only (98). Whether E_2_ acted directly on nonapeptide neurons in female gobies was not resolved, as the study did not investigate whether nonapeptide neurons express ERs. Oxt neurons in the POA of goldfish, *Carassius auratus*, are, at least based on immunohistochemical evidence, direct targets for estrogen actions, as they express the membrane estrogen receptor Gper1 (96) and are surrounded by radial glia, the only cells in the teleost brain expressing *cyp19a1b* and capable of producing neuroestrogens (136). While these studies provide evidence for effects of steroids on nonapeptide systems, future studies are warranted to delineate direct and indirect mechanisms of action, and whether effects are mediated by gonadal steroids and feedback regulation and/or local neurosteroids.

In sum, POA nonapeptide systems have been shown to be integrated into neuronal circuits involved in the regulation of the HPG axis in several teleosts. Additionally, recent evidence suggests that nonapeptide neurons are also responsive to sex steroids, suggesting the potential for endocrine feedback and/or modulation *via* neurosteroids. Additional studies investigating nonapeptide crosstalk with other hypothalamic regulators of the HPG axis are clearly warranted, as are careful studies investigating the direct or indirect regulation of hypothalamic nonapeptide systems to sex steroids, the critical endogenous reproductive signals.

#### 3.3.2 Nonapeptides exert direct hypophysiotropic effects on gonadotrophs in several species

In the goldfish, hypothalamic expression of *oxt* was found to peak seasonally in reproductively mature females (93). Pharmacological investigations demonstrate that hypothalamic induction of goldsfish *oxt* is dependent on GABAergic and dopaminergic signaling (137), in line with an integration of this nonapeptide system with seasonally-regulated HPG neurocircuitry in this species (92). Intraperitoneal injection of 1 μg/g body weight Oxt in sexually recrudescent female goldfish significantly increased circulating Lh by 167% 5h post-injection (86) with subsequent increases in circulating E_2_ 12h post injection (137). Unfortunately, Oxt-dependent stimulation of Lh release in sexually mature female goldfish was not investigated. Subsequent examination of potential direct effects of Oxt on gonadotrophs were investigated using primary goldfish dispersed pituitary cell cultures (87). In these preparations, Oxt significantly stimulated Lh release without affecting *lhb* or *fshb* subunit mRNAs, suggesting direct, transcription-independent stimulation of Lh release (87). These findings are in line with reported neuroanatomical evidence in other teleosts, such as the sailfin molly, *Poecilia latipinna*, the European bass, *Dicentrarchus labrax*, and the African sharptooth catfish*, Clarias gariepinus*, in which nonapeptidergic innervation of gonadotrophs has been reported (41, 48). Similar to Oxt, a stimulatory effect of Avp on Lh release has been demonstrated in at least two teleosts: in the sailfin molly 18 h pituitary incubation with Avp stimulated Lh synthesis and release, with lower dose- responsiveness and more consistent effects in male compared to female pituitaries (41). In the Asian stinging catfish, Avp, and to a much lesser extent Oxt and servatocin, stimulated *gpa*, *fshb* and *lhb* in pituitary cultures in a sex- and reproductive stage-dependent manner (24). The less potent effects of Oxt and servatocin where largely limited to *lhb* stimulation in pre-spawning females, with no effect on *fshb*. In female walking catfish, *Clarias batrachus*, pituitaries superfused with 20 nM Oxt for 1h displayed a significant decrease in Lhb staining reflective of increased Lh release was reported (138). In the ricefield eel, *Monopterus albus*, Oxt-stimulated Lh release from dispersed pituitaries *via* an Oxtra-activated IP_3_/Ca^2+^ pathway (81). In males of the Chanchita, *Cichlasoma dimerus*, Avp-stimulated gonadotropin secretion in single pituitary culture, with biphasic stimulation of Lh release at the lowest (0.1 μM) and highest (10 μM) concentration of Avp tested (54) and a stimulation of Fsh release at the highest Avp concentration tested.

These data establish a stimulatory role of nonapeptides on gonadotrophs, with sex-, reproductive stage- and species-dependent differences in potency and gonadotropin specificity. While it is important to keep in mind that observations are limited to only a few teleost species, data to date suggest that hypophysiotropic nonapeptide systems stimulate Lh release in teleost fishes similar to the situation reported in rodents and humans (139, 140).

#### 3.3.3 Endocrine and paracrine roles of nonapeptides in gonads

Both nonapeptides and their receptors have also been identified in ovaries and testes, and roles for nonapeptides in the regulation key gonadal functions *via* endocrine and paracrine signaling have been reported.

##### 3.3.3.1 Steroidogenesis

Effects of nonapeptides on male steroidogenesis have been reported in testicular cultures of the rainbow trout (141). Testosterone production stimulated by Avp and Oxt has been observed in immature but not mature testes *in vitro*. Exposure to Avp elicited a stronger maximal response in T production compared baseline production (6-fold) than Oxt (4-fold). The maximally active concentration of 100 nM Avp was furthermore found to augment dose-dependent Lh-stimulated T production, suggesting a synergistic role. In a similar study in chanchita, Avp-stimulated T production in testes incubated *in vitro* dose-dependently, reaching a significant, 2-fold increase at 50 nM (54). A limitation of these two studies is that the authors measured T, which is the prohormone for the more potent teleost sex steroids: 11-KT and E_2_. Unfortunately, nonapeptide receptor antagonists were not used in these experiments to probe specific receptor involvement. Since recent gene expression and *in-situ* hybridization data suggests a role for *avpr1aa* and *avpr2aa* receptors (**Table 3**), additional studies with teleost-validated antagonists are warranted to probe the molecular mechanistic basis involved in Avp-induced T production in testes.

Several studies have investigated the role of nonapeptides on ovarian steroidogenesis. While a stimulatory role for Oxt on circulating E_2_ has been reported in female goldfish *in vivo* (87), it was not investigated whether these effects are linked to prior increases in Lh (86) or mediated *via* direct action at the ovary. In contrast, a comprehensive study determining the role of Avp and Oxt on *in vitro* ovarian steroidogenesis at different seasonal reproductive developmental stages was conducted in the air-breathing catfish (142). Dose-dependent, biphasic stimulatory effects of Avp on E_2_ production for pre-vitellogenic ovarian tissue were reported. In contrast, Avp produced a dose- and time-dependent inhibition of E_2_ production in early postvitellogenic ovaries (142). In comparison, Oxt produced a low, yet significant, stimulation of E_2_ production without any dose effect in the previtellogenic ovaries, and a dose- and time-dependent inhibition like Avp in the early postvitellogenic ovary (142). The inhibitory effect of Avp on the E_2_ synthesis in the postvitellogenic ovary may be part of a trigger for the steroidogenic shift to decreased E_2_ in favour of synthesis of the maturation-inducing steroid (MIS) 17, 20β-dihydroxy-4-pregnen-3, 20-dione (17, 20β-DP) in air-breathing catfish (143). The MIS reinitiates oocyte meiosis up to the second metaphase (144).

Concurrent with the modulation of E_2_ synthesis, Avp and, to a lesser extent, Oxt also stimulated progesterone (P_4_) production (142). The Avp-stimulated increase in P_4_ was generally dose-dependent in pre- and early post-vitellogenic ovaries, reaching approximately a 30% increase in production compared to baseline at high doses (142). In late postvitellogenic ovaries, concentrations as low as 1 nM Avp induced a 60% increase over baseline concentrations (142). This effect was similar to that of hCG, and combined administration of 20 IU hCG and Oxt was found to be additive at least after 16 h incubation. Avp stimulated 17-hydroxyprogesterone (17-OHP_4_) synthesis in the previtellogenic phase ovaries following both 8 h and 16 h incubation (142). Avp stimulated the production of 17,20β-Dihydroxy-4-pregnen-3-one (17,20β-DP), which acts as maturation induced steroid in this species, about 2-fold more in the spawning phase than pre-spawning phase, similar to hCG. The stimulatory effect of Oxt was several-fold lower compared to Avp and occurred at higher concentrations (142). The combination of Avp and hCG elicited a cumulative effect on the 17,20β-DP level especially after 16 h of incubation in the spawning phase (142). The authors concluded that Avp was more potent than Oxt to stimulate the progestin pathway, and that Avp paralleled the actions of hCG. The finding that hCG and steroid hormones (E_2_, P_4_ and 17, 20β-DP) stimulate ovarian Avp production suggests a positive feedback loop (145) underscoring the functional significance of Avp in follicular growth, maturation, and ovulation. The stimulatory effect of Avp on ovarian P_4_ secretion is conserved as similar actions for AVP family peptides have been reported for chicken, mouse, and cow *in vitro* (146).

##### 3.3.3.2 Gametogenesis, gamete release and parturition

In the air-breathing catfish ovaries, Avp induces germinal vesicle breakdown (GVBD) and ovulation in a dose- and time-dependent manner (145). In this experiment, postvitellogenic follicles were co-incubated with Avp and an AVPR1 antagonist (deamino-Pen^1^, O-Me-Try^2^, Arg^8^ vasopressin), an AVPR2 antagonist (1-adamantane acetyl O-Et-D-Try^2^Val^4^, Abu^6^, Arg^8,9^ vasopressin), or both. GVBD, ovulation and 17,20β-DP concentration were inhibited or reduced by 92-94% after 24h co-incubation with both antagonists. The AVPR1 antagonist inhibited GVBD and ovulation by 82-83%, and the MIS concentration by 70%. The AVPR2 antagonist inhibited GVBD, ovulation and MIS concentration by 29%, 26% and 15%, respectively. The results show that the effects of Avp are mediated mainly by Avpr1 receptors with a minor role for Avpr2 receptors.

Prostaglandins (PGs) have a critical role in diverse aspects of reproduction in vertebrates (147). The cyclooxygenase inhibitor indomethacin can block MIS-induced final oocycte maturation and ovulation in yellow perch*, Perca flavescens*, and Atlantic croaker, indicating dependence on PGs (148, 149) The functional relationship between Avp and PGs was investigated in air-breathing catfish (150): Avp stimulated PGF_2α_ and PGE_2_ levels in a dose- and time-dependent manner *in vitro* and the effects were similar to that produced by hCG. Both Avp and hCG-induced stimulation of PG levels were inhibited by indomethacin, supporting involvement of cyclooxygenase. The Avp stimulation of PG levels was strongly inhibited by the AVPR1 receptor antagonist but not by the AVPR2 receptor antagonist. Indomethacin inhibited the Avp and hCG-induced GVBD and ovulation. Both PGF_2α_ and PGE_2_ stimulated GVBD and ovulation in a dose- and time-dependent manner and PGF_2α_ was more effective than PGE_2_. Taken together, these observations highlight a relationship between Avp and PGs, and their interaction in the control of oocyte maturation and ovulation.

Follicular or oocyte hydration is a phenomenon conspicuous and widespread in marine and catadromous fish eggs associated with follicular and oocyte maturation (FOM) and ovulation (151), and this process is retained to some extent in freshwater and anadromous fishes. Singh and Joy (152) reported a 23% rise in oocyte water content during the FOM and ovulation in *H. fossilis* with Avp eliciting a significant effect on oocyte water content, diameter, volume, osmolality, Na^+^K^+^ ATPase activity, Na^+^, K+, Mg^2+^, Ca^2+^ concentration, GVBD and ovulation, similar to hCG. The combination of Avp and hCG produced a higher effect. In a further study, Acharjee et al. reported that Avp regulates *aqp1ab*, (ovary-specific aquaporin 1ab) expression through an Avpr2 receptor (153, 154), which is linked to the cAMP-PKA pathway, similar to AVP in mammalian kidney tubules.

The involvement of nonapeptides in sperm release was first demonstrated in the killifish, *Fundulus heteroclitus*, in which fish and mammalian neurohypophyseal preparations as well as synthetic OXT initiated a spawning reflex response (155). The relative effectiveness of the nonapeptides to induce spawning reflex in the killifish was estimated to be the highest and equipotent for AVP and Avp, followed by Oxt and OXT (155). However, concentrations used in these original studies were high compared to physiological concentrations. In male African sharptooth catfish testes slices 30-min incubation with OXT (at 10 IU), but not Oxt, AVP, epinephrine, PGF_2α_, LH and pituitary extracts increased milt release (156). In male walking catfish, nonapeptides and their nanotube composites designed for slower release were tested for their efficacy to promote stripping of milt by abdominal massage (157). Both naked or nano-conjugated nonapeptides increased strippable milt concentrations without altering reproductive success of fertilized eggs and increased the expression of the steroidogenesis pathway enzymes *star*, *3bhsd*, *17bhsd*, *cyp17a1a*, ands *cyp11a1a* (157).

Regarding parturition in teleosts, nonapeptides have been reported to stimulate premature parturition in the guppy, *Poecilia reticulata*, an ovoviviparous teleost (82, 158). The injection of Avp, Oxt and PGs to guppy, *Poecilia reticulata*, a live-bearing teleost, induced premature parturition (157). Both Avp and Oxt stimulated *cox2* mRNA expression in guppy ovaries *in vitro*, which in the case of Avp, but not Oxt, translated into increased PG concentrations (82). Together this data suggests that the nonapeptide-dependent stimulation of premature parturition in guppies is mediated by PG. Interestingly, both Avp and Oxt exposure upregulated a guppy *oxtr* paralogue, suggesting interaction between ovarian nonapeptide systems in the guppy ovary (82). In zebrafish, a recent study investigating downstream effectors linked to a reduced ovulation phenotype observed in female chromosome 23 miR-200 cluster KO mutants showed that co-injection of hCG, Avp and Oxt, but not injection of synthetic human GnRH and LH analogues were able to partially rescue the phenotype (159). Together, this data suggests a Gnrh-independent role for Avp and Oxt in zebrafish ovulation. Nevertheless, early comparative evidence from teleost fishes has demonstrated that roles for nonapeptides on male and female spawning cannot be generalized in teleost fish and are possibly indirect following application of supraphysiological concentrations (160).

**Figure 4:**
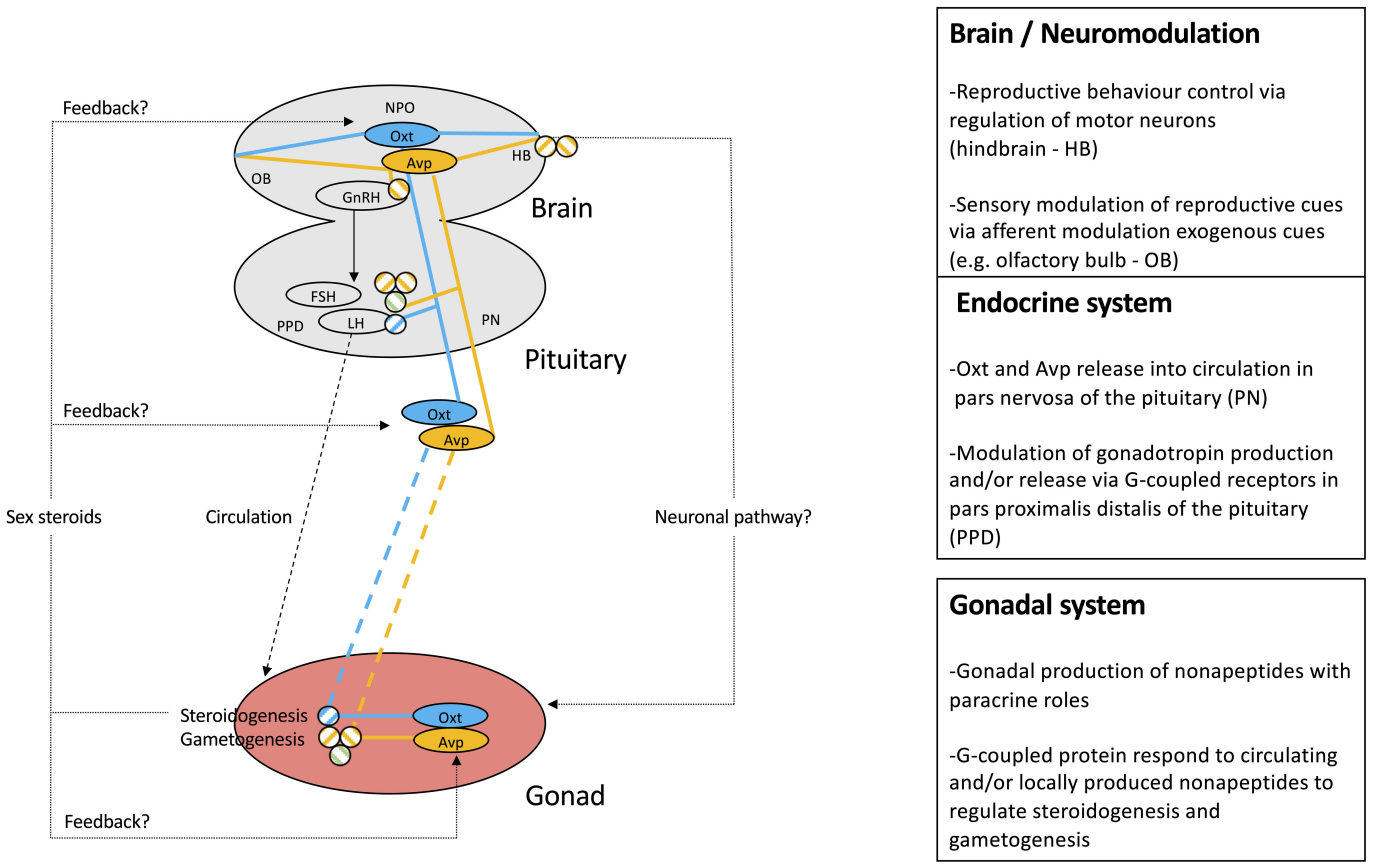
Schematic representation of mechanistic knowledge of nonapeptide roles in teleost reproduction. The Avp system is highlighted in yellow, while the Oxt system is represented in blue. Circled nonapeptides indicate sites of synthesis, while connecting lines represent neuronal, endocrine, and paracrine pathways. Specific receptor involvement in regulatory function of HPG axis components is symbolized as defined in **Figure 2**.

While we acknowledge a generally high degree of evolutionary conservation of nonapeptide genes and the gross (neuro)anatomical distribution of their expression, differences in reproductive function of nonapeptides between teleost species certainly exist. We have integrated the current state of knowledge of nonapeptides on central and HPG axis components of teleost reproduction in **Figure 4**. It is anticipated, however, that additional detailed comparative studies will uncover diversity of nonapeptide-dependent regulation of teleost reproductive physiology.

## 4 Translational aspects

As evolutionarily conserved systems regulating reproduction (3), research investigating roles of nonpeptides in teleost fish reproduction in detail have translational relevance in the areas of aquaculture and species conservation; both of which rely on methods informed by mechanistic understanding of reproductive physiology. As such, we anticipate that modulation of nonapeptide function in teleost species has significant potential to stimulate and possibly coordinate behavioural and endocrine processes necessary to promote reproduction for species in captivity (161). A third area of translational relevance is ecotoxicology. We review emerging evidence suggesting that neuroendocrine disruption of teleost nonapeptides may be linked to decreased reproductive success in teleost fishes, an ecologically meaningful endpoint (162).

### 4.1 Neuroendocrine disruption

In teleost fish, relatively recent studies have investigated Avp and Oxt nonapeptide systems as targets of different groups of aquatic contaminants. Histological studies of POA magnocellular neurons revealed that a six-month exposure of channel catfish, *Channa punctatus*, to inorganic mercury at a concentration of 10 µg/L resulted in smaller and less active Avp‐secreting neurons (163). Persistent organic pollutants have also been shown to affect nonapeptide systems in teleost fishes: in Atlantic Croaker, a four week daily dietary exposure (2 and 8 μg/g body weight) to the planar polychlorinated biphenyl congener 3,3′,4,4′-tetrachlorobiphenyl (PCB-77) significantly reduced hypothalamic expression of *avpr1a* mRNA and Avpr1a protein levels, as well as (co-localized) *gnrh1* mRNA levels in the brain (78).

Pharmaceuticals and plasticizers are other major environmental contaminants with reported effects on nonapeptide systems. Repeated intraperitoneal injections of pharmacological doses (5 µg/g) of the selective serotonin reuptake inhibitor and aquatic contaminant fluoxetine (FLX) significantly reduced *oxt* mRNA levels in female goldfish telencephalon and hypothalamus, an effect that was linked to reduced circulating E_2_ concentrations (85). Subsequent waterborne FLX exposure studies in both female and male goldfish revealed that *oxt* transcript abundance in the same tissues was not affected by a two- week exposure to FLX at 540 ng/L and 54 μg/L concentrations, but that the same two-week exposure to waterborne fluoxetine significantly diminished releaser pheromone PGF_2α_-induced increases in *oxt* mRNA (94, 164). Similarly, targeted gene expression analysis of zebrafish larvae acutely exposed to 50 and 500 ng/L FLX for 96 h (165) as well as transcriptomic screens of whole brains collected from a wild zebrafish population exposed to 100 μg/L FLX at a concentration for two weeks (166) identified *oxt* transcripts as being differentially expressed in FLX-exposed fish compared to unexposed control fish. Together, these studies reveal that the Oxt system is responsive to FLX at both early developmental and adult life-stages, raising the possibility of mediating organisational as well as activational effects. Repeated injection of pharmacological concentrations of 6 μg/g body weight FLX over a period of two weeks significantly reduced *avt* transcript abundance in giganto-, magno- and parvocellular neurons of the POA in male bluehead wrasses, an effect that correlated with decreased territorial aggression (167). The responsiveness of teleost nonapeptide systems to FLX corresponds to several observations in mammalian models (168, 169) and suggests an evolutionarily conserved serotonin-dependent regulation of these systems (87, 170, 171).

In Japanese medaka chronically exposed to environmentally relevant and high concentrations of waterborne metamphetamine for a period of 90 days, a dose-dependent, significant increase in whole brain *oxtr* mRNA and Oxt peptide were observed (172). Because both FLX and metamphetamine affect neurotransmitter systems and neuroactive contaminants may exert reproductive effects, at least in part, *via* nonapeptidergic systems, these findings support the concept of neuroendocrine disruption (173). In line with this interpretation, a meta-analysis of transcriptomic screens of the goldfish hypothalamus identified *oxt* as the single transcript affected by drugs modulating serotonergic, dopaminergic, and GABAergic systems, all of which have established roles in goldfish reproduction (137). Concurrent with previously described responsiveness of teleost POA nonapeptide systems to sex steroids, zebrafish chronically exposed to 1,10 and 30 μg/L Bisphenol A (BPA), a weakly estrogenic compound, as well as 1 μg/L E_2,_ exhibited complex dose-dependent and sex-specific effects on whole brain nonapeptide and nonapeptide receptor gene expression levels, which corresponded with alterations in social but not overall locomotor behaviour (174, 175). Similarly, developmental (2-5dpf) exposure to BPA and its replacement compound Bisphenol S (BPS) in the low μM range revealed non-linear alteration of *oxt* and Oxt protein abundance in association with quantifiable behavioural disruptions at 21dpf (176, 177). There is a need to study the involvement of nonapeptide systems in mediating organizational and/or activational effects of endocrine disrupting chemicals. This is an area understudied in teleost fish (178) compared to rodent models (179, 180), and will inform the possible development of teleost nonapeptides as functional biomarkers relevant to reproductive function in teleost fishes.

## 5 The state of the art and current limitations

Having critically reviewed the current state of knowledge regarding reproductive roles of teleost nonapeptides, we conclude by briefly discussing key insights and current limitations in the field. We furthermore suggest conceptual and technical approaches in the hope of stimulating collaborative research in the field.

1. It is well-appreciated that teleosts are champions of reproductive plasticity and diversity. There is a high degree of evolutionary conservation of nonapeptides and their gross (neuro)anatomical distribution. However, novel resources such as genomic sequences and detailed comparative investigation increasingly reveal areas of plasticity of teleost nonapeptide systems. A point in case is the nonapeptide receptor inventory, which exhibits differences between teleost fishes and other vertebrates, but, importantly, also within teleost fish lineages such as the otocephala and euteleosti (59). It will thus be important to address functional differences in nonapeptide receptor paralogue expression, regulation, and function to assess their potential roles in mediating reproductive functions in a reproductively diverse fishes.
2. While increasing comparative studies to delineate plasticity of nonapeptides in regulating reproduction in the diverse group of teleost fishes will be important, it will be equally crucial to comprehensively study the reproductive roles of nonapeptides within single teleost fish species. In reviewing the current knowledge of the role of nonapeptides in teleost reproduction, it has become clear that, apart from detailed studies in the Asian stinging catfish (50, 84) and, to a lesser degree, the canchita cichlid (54), few if any studies have investigated the role of nonapeptides across the different levels of the HPG axis within a single teleost species. The need for detailed studies within single fish species becomes even more clear when considering that research investigating behavioural and endocrine roles of nonpeptides in teleost reproduction has largely co-existed in isolation focussing on separate fish species. However, there is a need to consider (nonapeptide-regulated) courtship behaviour in the context of HPG axis function (165). Investigation of the salience hypothesis and possible roles for nonapeptides in the integration of reproductive cues are also warranted.
3. The recent generation of nonapeptide and/or nonapeptide receptor knock-out models in genetically-tractable model systems such as zebrafish (67) and medaka (64, 118) may hold particular promise. However, neither of these models have, to-date, been used to explore nonapeptide effects on the HPG axis. Receptor-specific knock-outs may furthermore prove fruitful to probe the role of virtually uncharacterized nonapeptide receptors such as *avtr2l* and generally circumvent possible specificity issues linked to the use of mammalian nonapeptide agonists and antagonists. Furthermore, GFP-reporter lines have been described in zebrafish, at least for Oxt (68, 69). While such lines represent powerful tools to investigate regulation and activation of central and peripheral nonapeptide systems in response to environmental and endogenous cues relevant to reproduction (35), few studies have been conducted in this area. Transgenic reporter lines may also allow endocrine disrupting) chemical screening for effects on nonapeptide systems. Adult-specific transgenic ablation studies and optogenetic approaches may in the future permit specific investigation of organisational and activational reproductive effects of the nonapeptides.
4. Previous co-localization experiments (43, 44) and more recent single-cell techniques (28) have demonstrated co-localization of nonapeptides with other neuropeptide regulators of reproduction in neuroendocrine neurons. Thus, future functional studies should investigate co-release and interactions between nonapeptides and co-expressed reproductive regulators to quantify potential combinatorial effects on courtship behaviour and HPG axis function.

## Author contributions

JM, DR, KS,RC, KJ, and VLT contributed to conception and design of the review. DR conducted in silico analyses. JM wrote the first draft of the manuscript. DR, KS, RC, KJ and VLT wrote sections of the manuscript. All authors contributed to manuscript revision, read, and approved the submitted version.

## Funding

The authors gratefully acknowledge the following funding sources: NSERC-Discovery Grants (Grant no. 147476 to JM, and to VLT), an NSERC-CGS-D (to KS), and partial funding from the Institute of Eminence of the Banaras Hindu University (to RC).

## Conflict of interest

The authors declare that the research was conducted in the absence of any commercial or financial relationships that could be construed as a potential conflict of interest.

## Publisher’s note

All claims expressed in this article are solely those of the authors and do not necessarily represent those of their affiliated organizations, or those of the publisher, the editors and the reviewers. Any product that may be evaluated in this article, or claim that may be made by its manufacturer, is not guaranteed or endorsed by the publisher.
